# Paving the career path for allied ophthalmic personnel

**Published:** 2020-12-31

**Authors:** Dhivya Ramasamy

**Affiliations:** 1 Senior Faculty: LAICO – Aravind Eye Care System, India.

**To build a formidable pool of trained allied ophthalmic personnel, retention, combined with the provision of opportunities, is vital.**

**Figure:**
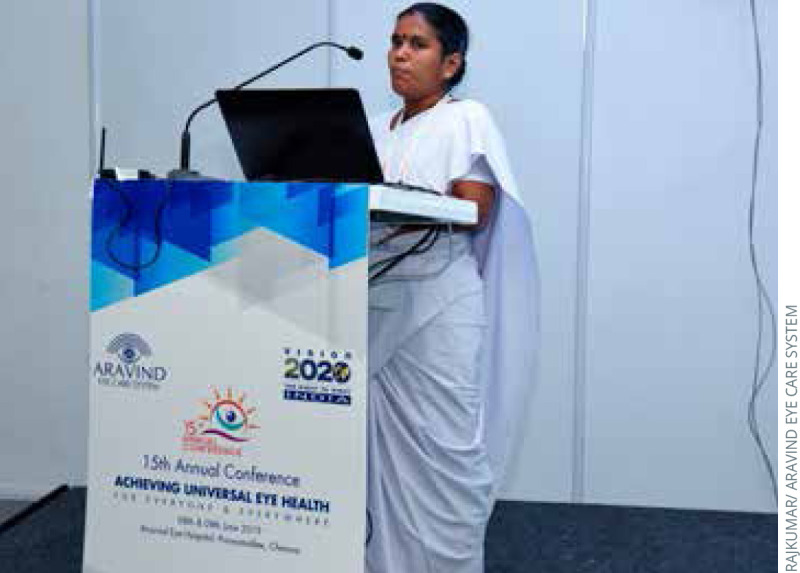
An AOP presents a paper at a national-level conference.

The role of allied ophthalmic personnel in the effective delivery of eye care has been highlighted repeatedly.^1^,^2^ This underscores the importance of national- level commitment, that recognises the levels and initiatives for effective training.^3^ Little has been done towards advancing these personnel and training them appropriately.

Creating growth and advancement opportunities is essential to make eye care an attractive career option, and to retain trained staff members. This article explores various ways in which organisations can structure the career growth opportunities for their allied ophthalmic personnel while making the most of their potential.

## Why must organisations create growth opportunities for AOPs?

It is important to offer opportunities for career development, not just to retain talented people, but also to ensure that they have opportunities to function to their fullest potential. Offering staff members a structured career path is attractive and can significally reduce the cost of recruitment and re-training. Also, the annual performance appraisals for AOPs help evaluate their performances and offer career development opportunities. These factors motivate their peers to give their best at work and help reinforce good performances.

**Case Study**

**Aravind Eye Hospital offers career advancement positions to AOPs who have more than three years work experience. Those who are exceptionally good at their work are recognised as ‘performers’. These staff members are also eligible for promotion as ‘supervisors’ (who take up leadership and supervisory roles) or as ‘tutors’ (who take up the role of senior trainers). The eligible candidates are assessed to see if they are fit to take up these positions - due diligence is carried out to ensure that they are suitable for positions with higher levels of responsibility. For instance, a 360-degree feedback is carried out to ensure that a staff member is eligible to become a supervisor. The selected candidates then receive additional training and orientation to fit into these new positions.**

**Also, the AOPs are provided support to pursue university programmes, such as a diploma in Ophthalmic Assisting, so that they can acquire formal recognition relevant to their work.**

## Different paths

Promoting staff to positions of higher responsibility would mean change in designations, along with added supervisory responsibilities.

- Promoting to more specialised areas: For instance, an AOP who typically attends to patients with cataract or refractive errors may be trained and promoted to assist in taking care of the patients with retinal disease or children with amblyopia.
- Offering opportunities to become trainers, coaches or mentors: This is relevant to the teaching institutions, where the teaching resources are precious and take time to build.
- Offering partial or full support to pursue higher education within their career track: This essentially helps in building a workforce with a high calibre combined with valuable experience. However, this may be affected by the availability of limited formal training programmes for AOPs in the region.

## Harness innovation

In addition to formal promotions and career pathways, opportunities can also be created by enhancing the enthusiasm of AOPs. These AOPs are generally efficient at their work and are keen to improve regularly. Providing opportunities for innovation and learning is an ideal way to keep them better engaged, while also harnessing innovation. Given the nature of their work, AOP staff are at the forefront of eye care and face many ground-level problems. Often, such situations also have good ideas in store. Organisations must provide opportunities for the staff to participate more actively by coming up with ideas and suggestions, allowing experimentation and tolerating failures. When combined with good opportunities, the passion of these professionals can lead to the generation of great ideas and solutions to everyday problems.

**Figure 2:**
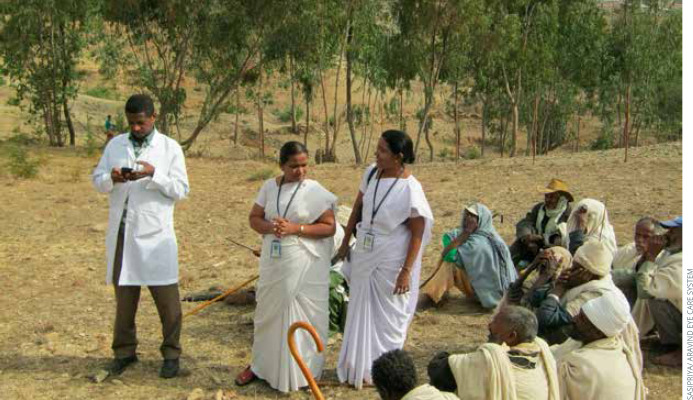
AOPs from Aravind providing guidance at an outreach camp in Ethiopia.

## Launch entrepreneurs

The organisations empower AOP staff with entrepreneur opportunities in the field. The eye hospitals encourage trained AOP staff to run vision centres in remote and rural areas, creating a win-win situation. By providing this opportunity, the hospitals can optimise resources that would be spent in reaching out to these communities. Often, AOP staff hail from these regions and have a better chance of acceptance and inclusion into these regions. If appropriately empowered, these entrepreneurs can continuously improve patient volumes, uptake of surgery and glasses, and ensure compliance to treatment.

## Academic opportunities

The AOPs can also identify new growth opportunities by becoming trainers and extending academic advancement. The training institutions can offer teaching roles to AOPs for internal and external training. The academic opportunities range from giving opportunities to participate in research, to encouraging presentations and participation in conferences, or may involve support to pursue higher education. The hospitals can also encourage AOPs towards participating as either learners or teachers in continuing professional education programmes.

## Make sure the transition is smooth

It is crucial to prepare the trainee for a new position in the right manner. Organisations often make the mistake of promoting candidates without preparing them for their new responsibilities. It is vital to ensure that proper training and orientation are given to the staff to help them succeed in their new roles. They may need to acquire new competencies, such as leadership skills. A clear job description of the new role helps in setting expectations for them. A certain amount of hand-holding during this transition period is also essential. All these must be included in the organisation's career advancement programme for AOPs.

## Points to note

Opportunities in career development alone cannot guarantee retention, as there are several contributing factors.^4^ While planning these opportunities for AOP staff, it is equally important to consider internal and external contexts. Meticulous planning and due diligence must be applied in choosing the right candidates for advancement. A formal performance appraisal, combined with career development, can be a great incentive and motivation for eye care workers.^5^
